# Characterization and Optimization of Polymeric Bispicolamine Chelating Resin: Performance Evaluation via RSM Using Copper in Acid Liquors as a Model Substrate through Ion Exchange Method

**DOI:** 10.3390/molecules27217210

**Published:** 2022-10-25

**Authors:** Kowit Suwannahong, Chadrudee Sirilamduan, Anat Deepatana, Torpong Kreetachat, Surachai Wongcharee

**Affiliations:** 1 Department of Environmental Health, Faculty of Public Health, Burapha University, Chonburi 20131, Thailand; 2 Department of Business Management, Faculty of Management Science, Ubon Ratchathani University, Ubon Ratchathani 34150, Thailand; 3 Department of Chemical Engineering, Faculty of Engineering, Burapha University, Chonburi 20131, Thailand; 4 Department of Environmental Engineering, School of Energy and Environment, University of Phayao, Phayao 56000, Thailand; 5 Field of Environmental Engineering, Faculty of Engineering, Mahasarakham University, Mahasarakham 44150, Thailand

**Keywords:** ion exchange, copper, chelating resin, Dowex-M4195

## Abstract

Advanced technologies of electronics industries have led to environmental contamination concerns, especially waste print circuit boards containing a very high concentration of copper (II) ions, which can be discharged in wastewater containing many contaminated metals. A low pH is a necessity for treating industrial wastewater containing heavy metals to meet engineering process design. A novel polymeric bispicolamine chelating resin, Dowex-M4195, was applied as an alternative for investigating the behavior of copper (II) in acidic solution via an ion exchange method in a batch experiment system. Characterization of physical and chemical properties before and after ion exchange were also explored through BET, SEM-EDX, FTIR and XRD. Response surface methodology was also applied for optimization of copper (II) removal capacity using design of experiment for selective chelating resin at a low pH. The results indicate that H^+^ Dowex-M4195 chelating resin had a high-carbon content and specific surface area of >64% and 26.5060 m^2^/g, respectively. It was predominantly macropore porous in nature due to the N_2_ gas adsorption isotherm and exhibited type IV with insignificant desorption hysteresis loop of H1-type. It was spherical and cylindrical. After the ion exchange process of copper (II)-loaded H^+^ Dowex-M4195, the specific surface area and total pore volume decreased by about 17.82% and 5.39%, respectively, as compared to H^+^ Dowex-M4195. Hysteresis loop, isotherm and pore size distribution were also similar. Regarding the functional group, the surface morphology and crystalline structures of H^+^ Dowex-M4195 showed copper (II) compound based on the structure of chelating resin that confirmed effective ion exchange behavior. The design of optimization indicated that copper (II) removal capacity of about 31.33 mg/g was achieved, which could be obtained at 6.96 h, pH of 2 (a desirable low pH), dose of 124.13 mg and concentration of 525.15 mg/L. The study indicated that the H^+^ Dowex-M4195 (which is commercially available on the market) can successfully be applied as an alternative precursor through the ion exchange method for further reuse and regeneration of the copper (II) in the electronic waste industries and other wastewater applications needed to respond the policy of biocircular green economy in Thailand.

## 1. Introduction

Various types of chelating resins are well known and widely used in several applications for water and wastewater treatment plants, especially in industrial estates for removing metals selectively via commercially available ion exchange processes. In recent years, different functional groups of chelating resin were widely used for base metal purification and separation [1] through the ion exchange process, especially for rare earth elements such as Amberlite XAD-4 (styrene divinyl benzene copolymer) [2], Dowex-M4195 and Dow-4196 (bis-picolylamine) [3], Lewatit TP 207 and SIR-300 (iminodiacetate) [4,5], SIR-500 (amino phosphonic) and Dowex XUS43605 (hydroxypropylpicolylamine) [6,7]. These chelating resins were applied for extraction of various metals such as Pb(II)/lead, Cu(II)/copper, Cd(II)/cadmium, Zn(II)/zinc, La(III)/lanthanum, Ni(II)/nickel, Cr(III, IV)/chromium, Fe(II)/iron and Co(II)/cobalt ions [8,9] from aqueous solutions and especially in electronics waste applications at a low pH. As mentioned above, the literature reported that Dowex-M4195 chelating resin showed the best results in removing/ion exchange Cu(II) at a low pH of less than 2 [10] as compared to other functional groups of chelating resin. However, the selective chelating resins in terms of ionic form such as Na^+^ and H^+^ are still limited regarding information in the literature for removing of Cu(II) in the electronics industries. In addition, alternative approaches for extraction of Cu(II) were mentioned from the point of view of environmental friendliness and economy such as using microorganisms as leaching agents for the removal and recovery of metal from electronic waste. Therefore, using microorganisms has become an alternative choice for extraction of metal from industrial wastewater through an ion exchange process.

Currently, the rapid expansion/advanced technologies of electronics industries have led to environmental contamination concerns, especially waste print circuit boards (PCBs)/E-waste containing a very high concentration of Cu(II) ions, which can be discharged in wastewater containing many contaminated metals [11]. PCBs are electronic devices (≥1.4 million tons discarded/year) [12] that contain metals (40%), polymer (30%) and ceramics (30%) [11], and the Cu concentration is higher than in nature in wastewater sources as compared with other metal types. There have been many studies investigating the use of different physicochemical methods in removal, reuse and recovery of copper from electronic waste, such as adsorption [13,14,15,16], incineration [17], landfill dumping [18], advanced/chemical oxidation [19] and precipitation [20]. Although such approaches could minimize the effect of electronics waste, they nonetheless have limitations [21] and lack cost-effectiveness. On the other hand, Igiri et al. [22] reported bioleaching that is an eco-friendly technology as a circular economy for extracting valuable divalent metals such as Cu(II) ions from electronic waste [23,24,25]. It can be seen that E-waste is an important source of Cu that can be utilized as artificial ores using chemical precipitation, solvent extraction, adsorption and ion exchange processes for reuse and recovery of divalent metal from E-waste wastewater. The ion exchange approach is an alternative technology for removal and recovery of the leaching metal from E-waste [26,27] via chelating resin of bispicolylamine functional groups such as Dowex-M4195 that provide efficient ion exchange between a liquid and solid phase of Cu(II) and resin. However, characterization and optimization of bispicolamine Dowex-M4195 chelating resin performance evaluation of ion exchange via response surface methodology have not been investigated and a low pH is a necessity for electronic waste removal from wastewater. A few studies and detailed knowledge have been reported on the physical properties of Dowex-M4195 before and after ion exchange with Cu(II) leaching by acetic acid (a low pH < 2).

Hence, this work aimed to investigate the behavior of Cu(II) adsorbed/ion exchanged onto Dowex-M4195 chelating resin bispicolylamine functional groups for sodium and hydrogen form in a batch adsorption experiment. The characterization of physical and chemical properties before and after ion exchange was also explored. Response surface methodology (RSM) was also applied for optimization of Cu(II) removal capacity via an ion exchange process in design of experiment (DOE) for further reuse and recovery of selective chelating resin.

## 2. Results and Discussion

### 2.1. SEM Images of H^+^ Dowex-M4195 and Cu(II)-Loaded H^+^ Dowex-M4195 Chelating Resin

**Figure 1:**
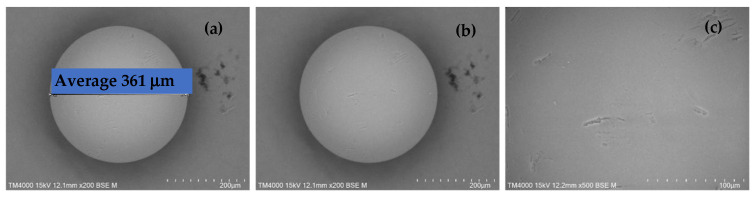
High-magnification SEM images of H^+^ Dowex-M4195 chelating resin at a magnification of ×200; (**a**) average dimensions, (**b**) spherical form and (**c**) the surface of resin.

**Figure 2:**
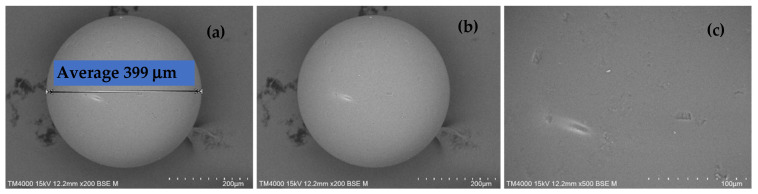
High-magnification SEM images of Cu(II)-loaded H^+^ Dowex-M4195 chelating resin at a magnification of ×200; (**a**) average dimensions, (**b**) spherical form and (**c**) the surface of resin.

The morphology and structure of obtained samples of H^+^ Dowex-M4195 and Cu(II) loaded onto H^+^ Dowex-M4195 were investigated using SEM. As shown in Figure 1 and Figure 2, SEM images illustrate that both H^+^ Dowex-M4195 and Cu(II)-loaded H^+^ Dowex-M4195 had a spherical form. The external surface was rough and not smooth with a large number of macropores (Physical properties of H^+^ Dowex-M4195 and Cu(II)-loaded H^+^ Dowex-M4195 obtained from a different method of BET and BJH methods), however, Cu(II) loaded onto H^+^ Dowex-M4195 was smoother than with normal H^+^ Dowex-M4195. In addition, it can be seen that the dimensions were different as shown in Figure 1 and Figure 2 (SEM images of H^+^ Dowex-M4195 chelating resin, 361 μm, 0.631 mm) and Figure 1 (SEM images of Cu(II) loaded onto H^+^ Dowex-M4195 chelating resin, 399 μm, 0.399 mm). After the ion exchange process, the demission size of H^+^ Dowex-M4195 increased by around 9.52% with a high-magnification SEM inset scale bar of TM4000 15 kV 12.1 mm × 200 BSE M. It can be concluded that H^+^ Dowex-M4195 performed well for ion exchange between Cu(II) in the ion exchange process.

### 2.2. Leica Microscope Image Analyses of H^+^ Dowex-M4195 and Cu(II)-Loaded H^+^ Dowex-M4195 Chelating Resin

**Figure 3:**
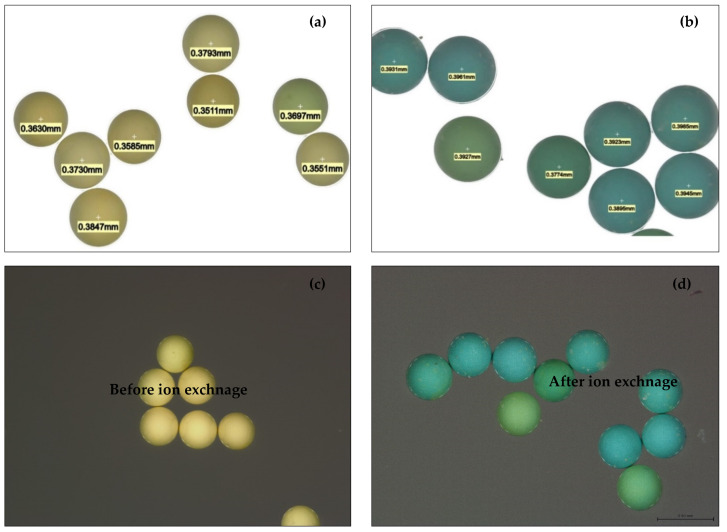
Leica microscope images: (**a**) dimension range of H^+^ Dowex-M4195, (**b**) dimension range of Cu(II)-loaded H^+^ Dowex-M4195, (**c**) H^+^ Dowex-M4195 chelating resin, (**d**) Cu(II)-loaded H^+^ Dowex-M4195.

To confirm the investigation of the ion exchange process, a stand-alone Leica microscope was applied to depict the size and color of the samples as shown in Figure 3. It can be seen that the H^+^ Dowex-M4195 had a green-yellow color with a demission of around 0.3511–0.3793 mm (Figure 3a). After the ion exchange process, Cu(II)-loaded H^+^ Dowex-M4195 chelating resin had dimensions of 0.3774–0.3985 mm (Figure 3b) with a blue-green color. This can be seen in the SEM images (Section 2.1 SEM images of H^+^ Dowex-M4195 and Cu(II)-loaded H^+^ Dowex-M4195 chelating resin) depicted in this range. Figure 3c,d visually confirmed that Cu(II) adsorbed onto the surface of the chelating resin before and after ion exchange.

### 2.3. Elemental Analysis of Compositions of H^+^ Dowex-M4195 and Cu(II)-Loaded H+ Dowex-M4195 Chelating Resin

**Figure 4:**
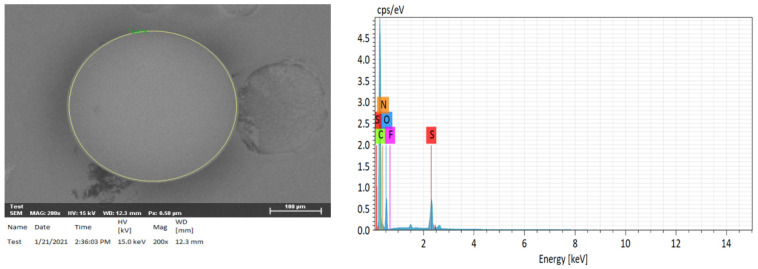
SEM-EDX image of H^+^ Dowex-M4195 chelating resin.

**Figure 5:**
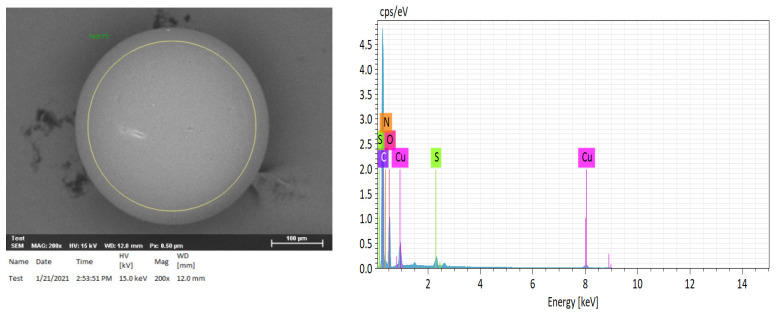
SEM-EDX image of Cu(II) loaded onto H^+^ Dowex-M4195 chelating resin.

**Figure 6:**
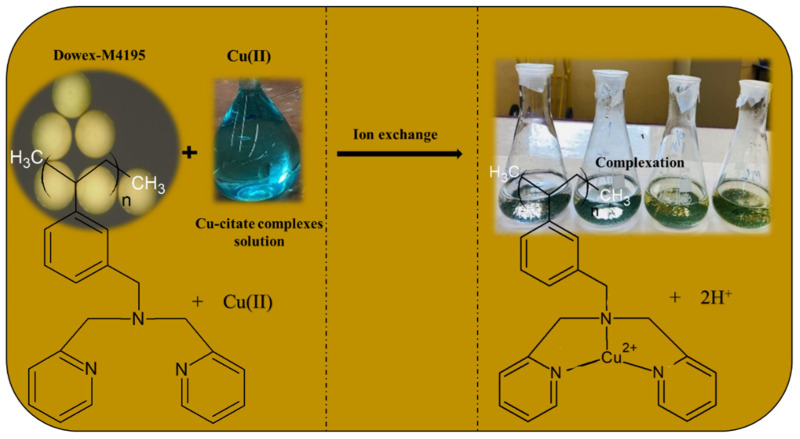
Possible mechanism of ion exchange of H^+^ Dowex-M4195 chelating resin with Cu(II).

Figure 4 and Figure 5 present the SEM-EDX results illustrating the images with the elemental composition of H^+^ Dowex-M4195 and Cu(II) loaded onto H^+^ Dowex-M4195 chelating resin, which were used for physical morphology and approximation of the elemental compositions of before and after materials. The EDX spectra before and after ion exchange revealed the elements C, O, N, S, F and Cu of both chelating resins. Figure 4 indicates that SEM-EDX image analysis of H^+^ Dowex-M4195 chelating resin shows C (64.50%), O (20.26%), N (11.77%), F (0.38) and S (3.01%) as shown in Table 1. It shows no Cu(II) adsorbed or ion exchange bound onto the surface of H^+^ Dowex-M4195 chelating resin, while Cu(II)-loaded H^+^ Dowex-M4195 demonstrated that the ions of Cu(II) were exchanged onto the surfaces of chelating resin at about 4.66 percent. It could be summarized that ion exchange was possibly the main mechanism/process for Cu(II) adsorption as presented in Figure 6 (RH_n_ + Cu^2+^ ⇄ R-Cu^2+^ + nH^+^) [28].

### 2.4. Nitrogen Adsorption–Desorption Isotherms of H^+^ Dowex-M4195 and Cu(II)-Loaded H^+^ Dowex-M4195 Chelating Resin

**Figure 7:**
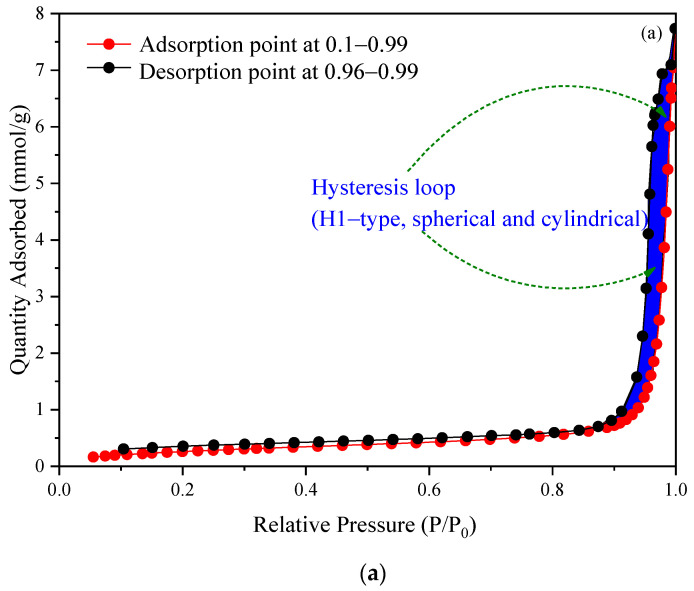

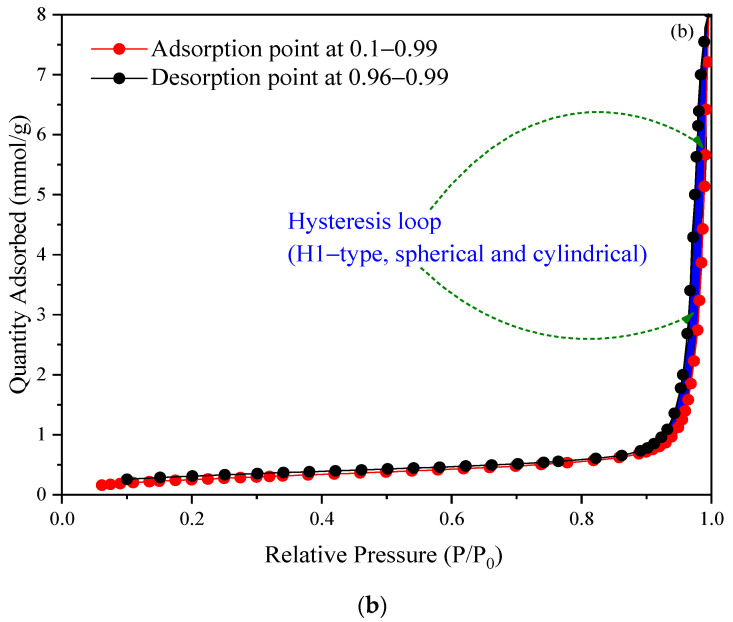
N_2_ adsorption–desorption isotherms of H^+^ Dowex-M4195 chelating resin: (**a**) before ion exchange and (**b**) after ion exchange.

Figure 7 presents the shape adsorption–desorption isotherms of H^+^ Dowex-M4195 chelating resin before and after Cu(II) removal via the ion exchange process. The bulk of N_2_ was absorbed at a constant pressure of about 101.3250 kPa with an equilibration interval of about 10 s and a sample density of 1.000 g/cm^3^. The quality of N_2_ adsorbed (mmol/g) is presented as a function of the relative pressure (P/P_0_) at 77.3 K, where the adsorption point is at 0.1–0.99 and the desorption point is at 0.96–0.99 P/P_0_. The adsorption isotherm before and after Cu(II) removal was close to type IV [29] following the International Union of Pure and Applied Chemistry (IUPAC) classification guidelines [29,30,31]. The physical adsorption isotherms both before and after exchanging did not exhibit a saturation as type I (monolayer adsorption) [32], II (multilayer adsorption) [33] or III (adsorbate–adsorbate attractive interactions, convex) [34,35]. However, type IV was well defined due to the N_2_ adsorption–desorption curves showing the insignificant desorption hysteresis loop (H1-type, spherical and cylindrical) [36], suggesting that the samples had a mesopore volume or uniform mesoporous/macropore structure. Moreover, the desorption hysteresis loop illustrated a very clear curve with a relative pressure (P/P0) over 0.90, indicating that H^+^ Dowex-M4195 chelating resin had a structure of macropores as shown in Table 2. The resin had a very low total pore volume that was 0.2698 cm^3^ g^−1^ (H^+^ Dowex-M4195) and 0.2372 cm^3^ g^−1^ (Cu (II)-loaded H^+^ Dowex-M4195) as shown by the different size of the desorption hysteresis loop in Figure 7, where a small loop indicates the mass of Cu(II) was adsorbed/ion exchanged to the pore or the surface of the chelating resin used.

### 2.5. Physical Properties of H^+^ Dowex-M4195 and Cu(II)-Loaded H^+^ Dowex-M4195 Chelating Resin

Two approaches were used to explore physical porous properties: Brunauer–Emmett–Teller theory (BET) and Barrett–Joyner–Halenda (BJH) methods, which have been widely used for specific surface area, total pore volume and pore size distribution (PSD) of materials and the results obtained in this work are presented and compared in Table 2. BET and BJH adsorption cumulative specific surface area of pores of 17–3000 Å range was investigated. It can be seen that the H^+^ Dowex-M4195 had a comparably higher specific surface area (26.5060 m^2^/g) than the Cu(II)-loaded H^+^ Dowex-M4195 (21.7810 m^2^/g) for the BET method with about a 17.82% difference. The BJH method result was the same as that of the BET method with a difference of about 14.36% whereas the specific surface areas of H^+^ Dowex-M4195 and Cu(II)-loaded H^+^ Dowex-M4195 were about 28.2635 and 24.2043 m^2^/g, respectively. The specific surface area can be decreased simply due to the Cu(II) adsorbed/ion exchange onto the surface of the H^+^ Dowex-M4195 chelating resin used. However, the effects on the surface area and other physical properties, such as functional groups and crystal structure of H^+^ Dowex-M4195 chelating resin, are considered in the next section.

### 2.6. Pore Size Distribution and Pore Volume of H^+^ Dowex-M4195 Chelating Resin

**Figure 8:**
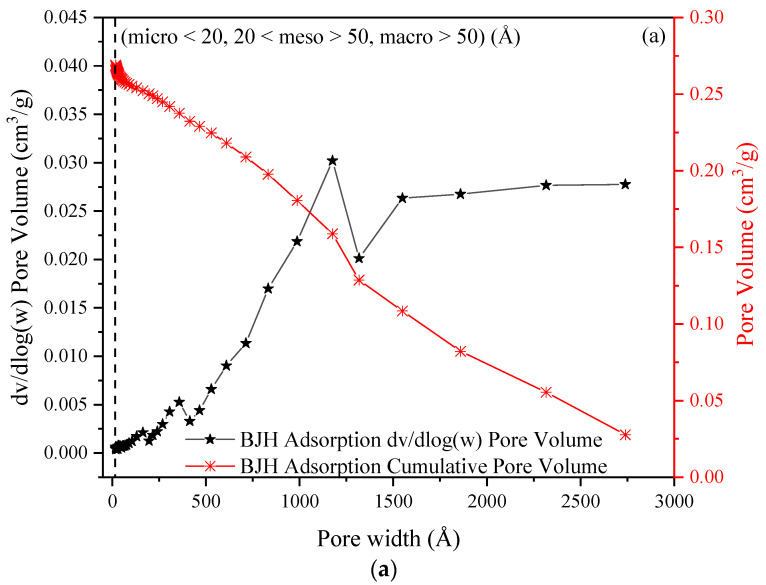

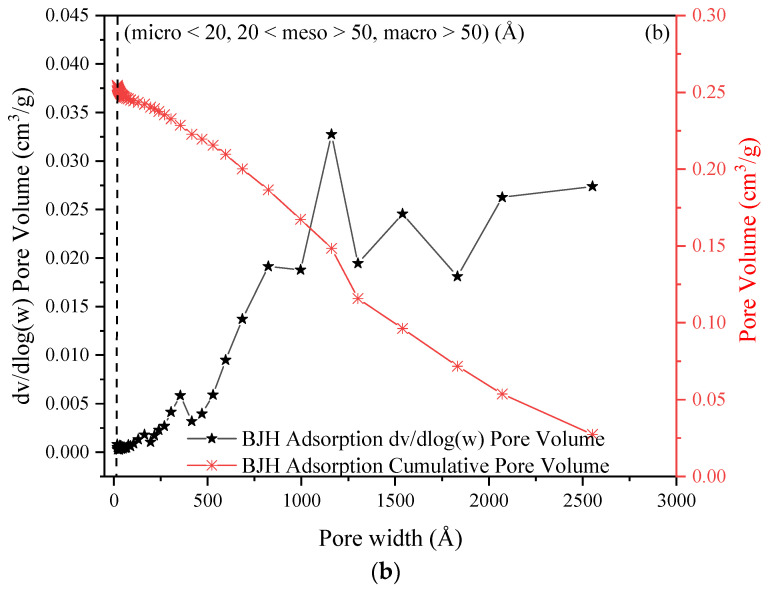
BJH adsorption pore volume of H^+^ Dowex-M4195 chelating resin: (**a**) before ion exchange and (**b**) after ion exchange process.

Total pore volume and pore size (17–3000 Å width) distribution at P/P_0_ = 0.9950 relative pressure were investigated. The pore size distribution of materials obtained was determined using BJH methods as shown in Figure 8, in which results both before and after ion exchange showed that H^+^ Dowex-M4195 chelating resin consisted mainly of a macroporous structure with ASTM standard size (micro < 20, 20 < meso > 50, macro > 50). It can be seen that the average pore width of H^+^ Dowex-M4195 chelating resin (380.7060 Å) was less than Cu(II)-loaded resin (420.6690 Å). In addition, total pore volume also confirmed that H^+^ Dowex-M4195 chelating resin was fully ion exchanged with Cu(II) owing to the pore volume decrease of about 5.39% as presented in Table 2. These results indicated clearly that the H^+^ Dowex-M4195 chelating resin had exchanged the ions of Cu(II) onto the surface and was macroporous in nature.

### 2.7. Functional Groups before and after Ion Exchange Study of the H^+^ Dowex-M4195 Chelating Resin 

**Figure 9:**
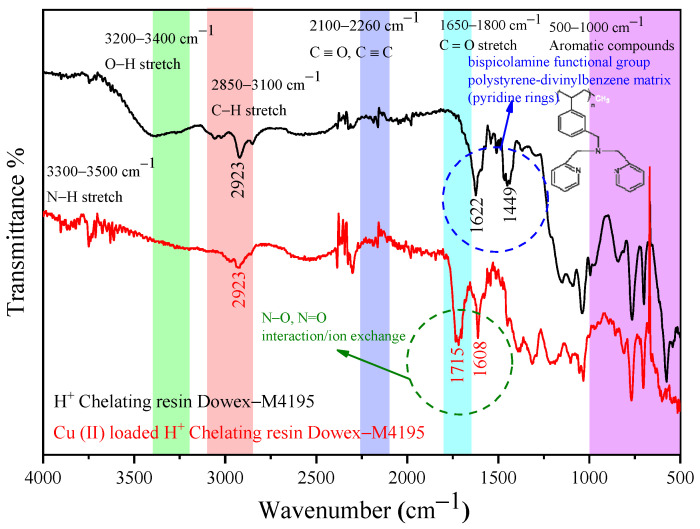
FTIR spectrum before and after Cu(II) was loaded onto H^+^ Dowex-M4195 chelating resin.

FTIR analysis was used to determine changes in the structure of surface functional groups before and after the ion exchange process for the H^+^ Dowex-M4195 chelating resin at the wavenumber range of 400–4000 cm^−1^. The functional group has a significant role in ion exchange behavior due to its largely regulating specific affinities toward various metal pollutants in water and wastewater sources. Figure 9 presents the plotted FTIR spectra for before and after the ion exchange process of the resin. It shows the strong absorption bands at 1449 and 1622 cm^−1^ (H^+^ Dowex-M4195 chelating resin) indicating the bispicolamine functional group or polystyrene–divinylbenzene matrix (pyridine rings) functional groups [37] of the chelating resin before the ion exchange process. The peaks at 1715 and 1608 cm^−1^, which appeared in the spectra after the ion exchange process (Cu(II)-loaded H^+^ chelating resin Dowex-M4195), had been transformed into new broad absorption peaks. It indicated that those peaks may be attributed to the interactions/occurrence/combination [38,39] between nitrogen (protonated) and Cu(II) (divalent metal) [10]. In addition, the stretching vibration of the alkane groups (CH_3_, CH_2_ and CH, 2 or 3 bands) was exhibited at around 2923 cm^−1^ and other fundamental peaks before and after the ion exchange process were almost the same. It is suggested that the ion exchange process of Cu(II) corresponds to the stretching vibration of Cu (II)=O or Cu (II)-O and showed predominantly N=Cu, N-Cu, N-O or N=O groups.

### 2.8. Crystal Structure before and after Ion Exchange Study of the H^+^ Dowex-M4195 Chelating Resin

**Figure 10:**
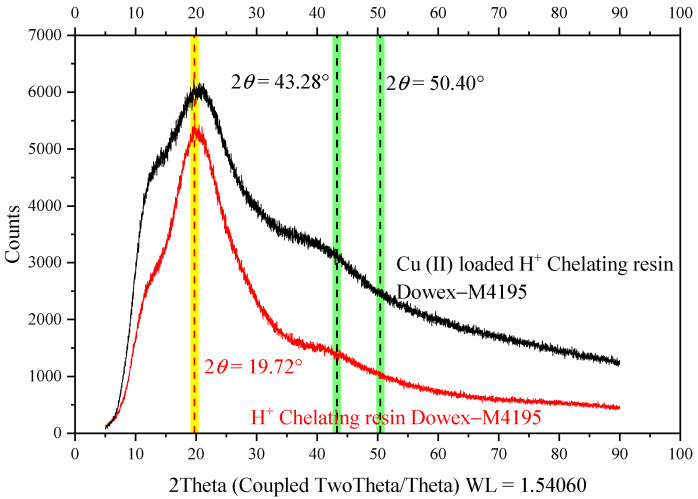
Typical XRD patterns (experimental pattern) before and after the ion exchange process.

The crystal structure before and after the ion exchange process of the resin was investigated using X-ray powder diffraction by measuring the intensity of radiation reflected at various angles from 5° < 2*θ* < 90° with registering of Cu K^α^ at 1.54060 as shown in Figure 10. The maximum diffraction peaks were observed at 2*θ* = 19.72°, which is the same as reported in the literature [40]. The figure shows that the intensity of the peak detected for Cu(II) loaded onto H^+^ Dowex-M4195 chelating resin was higher than that for H^+^ Dowex-M4195 chelating resin, whereas the maximum diffraction was related to the crystalline region. Cu(II) loaded onto H^+^ Dowex-M4195 chelating resin showed two broad peaks at 43.28° and 50.40° [41] which can be indexed as the (111) and (200) [42] plane reflections, respectively, which indicated the cubic lattices of the copper. The diffraction peak (hairy appearance) results also showed the presence of cubic lattices of copper crystalline structure in the H^+^ Dowex-M4195 chelating resin after the ion exchange process that increases the size of chelating resin as has been explored in the literature [43]. 

### 2.9. RSM and Model Fit for Cu(II) Removal onto H^+^ Dowex-M4195 Chelating Resin

Thirty observed responses of *q_e_* (mg/g) were obtained from the design RSM-CCD matrix (non-center point 24 and center point 6). Obtained *q_e_* responses were utilized to generate the empirical model terms using RSM to formulate the model summary statistics for Cu(II) loaded onto H^+^ Dowex-M4195 chelating resin. The adsorption capacity/removal capability (*q_e_* (mg/g)) of H^+^ Dowex-M4195 chelating resin was investigated as a quadratic model computed using software. We focused on the model with the highest R-squared value, adjusted R-squared or predicted R-squared and order polynomial where the additional terms are significant and the model is not aliased, as presented in Table 3. 

Analysis of variance (ANOVA) suggested a quadratic model, which was related to four actual variable factors of Time (*x*_1_), pH (*x*_2_), Dose (*x*_3_), and Conc. (*x*_4_), as illustrated by Equation (4). Negative and positive signs shown in the coded equation model terms indicate antagonistic and synergistic effects.

*q_e_* (mg/g) = 40.65 + 12.41*x*_1_ + 0.6341*x*_2_ − 1.99*x*_3_ + 12.75*x*_4_ + 0.3950*x*_1_*x*_2_ − 1.19*x*_1_*x*_3_ + 11.52*x*_1_*x*_4_ − 0.0050*x*_2_*x*_3_ + 0.3950*x*_2_*x*_4_ − 1.56*x*_3_*x*_4_ − 20.73*x*_1_^2^ + 1.32*x*_2_^2^ + 7.89*x*_3_^2^ − 17.66*x*_4_^2^ (1)

Table 4 demonstrates the ANOVA of the regression model for Equation (4). F-value and *p*-value statistical significance were used to indicate if the regression model was significant using Student’s *t*-test. It can be seen that the model F-value used (29.13) implies the quadratic model is substantial whereas the *p*-value was less than 0.05 (the error probability <0.0001) and the lack of fit (F-value = 1.76) was not significant relative to the pure error [44,45,46]. The higher R-squared (0.9645, close to 1) also confirmed the order polynomial model of Equation (4) that Cu(II) loaded onto the H^+^ Dowex-M4195 chelating resin can be explained by the selected quadratic model which was computed using the software system. In addition, the fit statistic of the adjusted R-squared (0.9314) and predicted R-squared (0.8553) had a difference of less than 0.2, indicating that the model *q_e_* is in reasonable agreement and significant for predicting the response of maximum ion exchange capability Cu(II) removal through the ion exchange approach. 

Table 5 presents code and actual variable factors related to observed and predicted responses *q_e_* via a batch ion exchange process of thirty runs. Observed and predicted responses *q_e_* were compared in terms of the residual plots that are detailed in the next section. 

**Figure 11:**
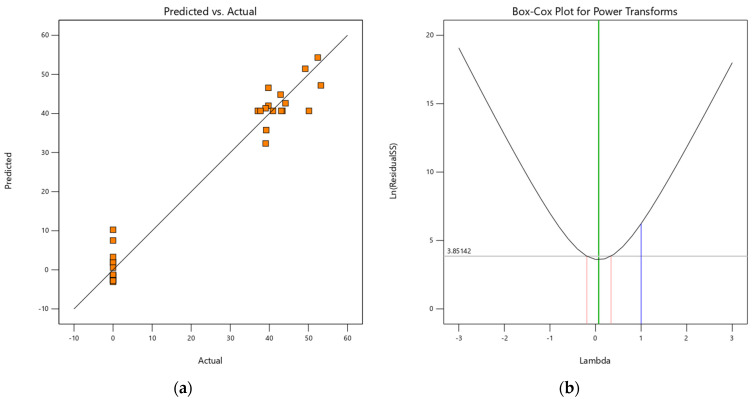
(**a**) Actual values vs. predicted values and (**b**) Box–Cox plot power transforms for Cu(II) loaded onto H^+^ Dowex-M4195 chelating resin from the selected quadratic model terms.

The plots of actual value vs. predicted values and Box–Cox plot power transforms for Cu(II) loaded onto H^+^ Dowex-M4195 chelating resin obtained from the quadratic model are presented in Figure 11a,b. It can be seen in Figure 11a that this scenario might be considered to provide adequate confidence in the experimental batch system. It was found that the distribution characteristics of the data were distributed near the regression line, indicating that this quadratic model term is significant whereas the F-value < 0.005 and *p*-value < 0.00001 (Table 4) were not. Box–Cox Plot for Power Transforms statistical tool was also used for determining the precision of the selected quadratic model terms. It found that the power of lambda was between 0 and 1 (Figure 11b), indicating the extreme acceptance percentage of the quadratic model term or main hypothesis. On the other hand, it can be concluded that the regression model terms fit with reasonable precision between the observed values and the predicted values from the selected quadratic model. 

**Figure 12:**
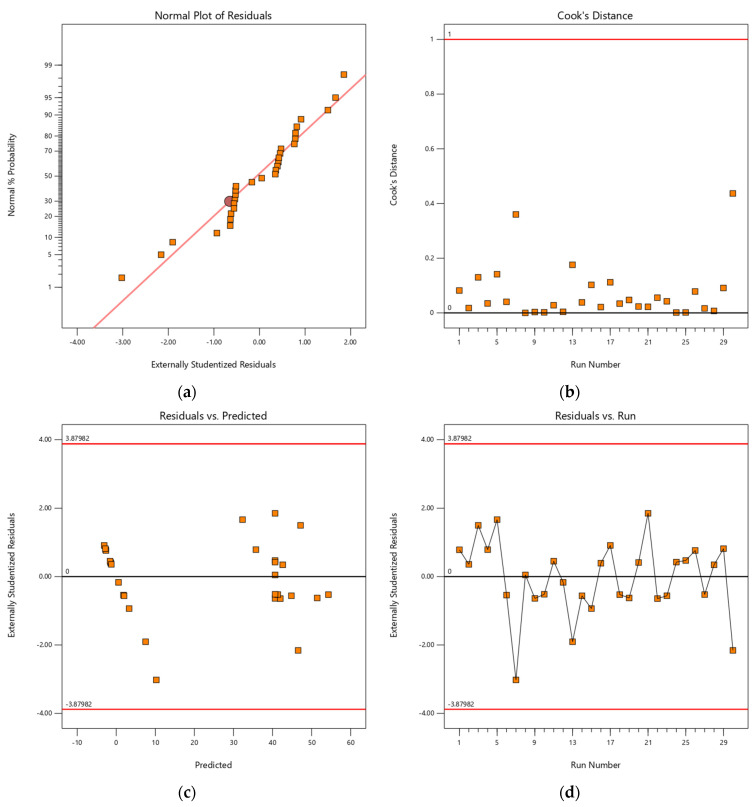
(**a**) A normal plot of residuals, (**b**) Cook’s distance, (**c**) residual vs. predicted and (**d**) residual vs. run plots for Cu(II) loaded onto H^+^ Dowex-M4195 chelating resin from a selected quadratic model term.

Figure 12a–d denote the normal plot of residuals, Cook’s distance, residual vs. predicted and residual vs. run plots, respectively. Figure 12a implies that the normal probability plots produced a good correlation with a straight line that assured the model term had significance. Figure 12b illustrates Cook’s distance plot; it can be observed that each run number was lower than the red straight-line plot (<1), indicating strong influence of fitted values of the selected quadratic model. In addition, other statistical tools such as residual vs. predicted and residual vs. run plots were also used to confirm the selected model. It can be seen that distributed data points did not detect outliers of the red straight-line plots (3.87982). It is suggested that the selected quadratic model is accurate and adequate for the selection of copper removal via an ion exchange process with the selected chelating resin for sustainable water reuse and can be applied in engineering water treatment process design. 

**Figure 13:**
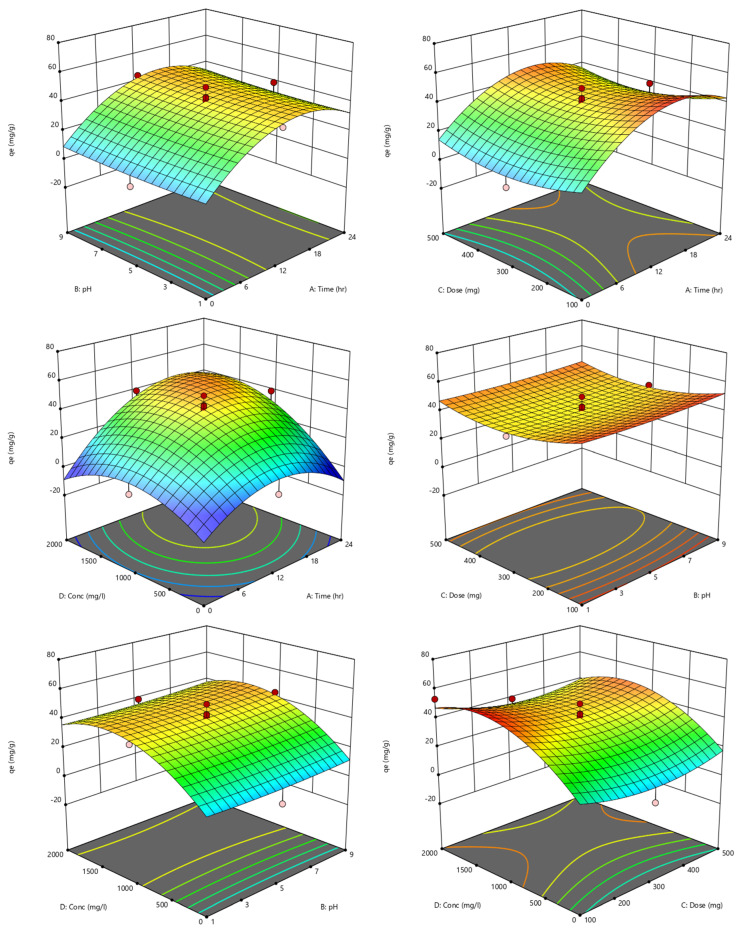
The 3D dimension images of Cu(II) removal efficiency onto H^+^ Dowex-M4195 chelating resin from the selected quadratic model related to the pH, Time, Dose, Conc. and *q_e_*.

**Figure 14:**
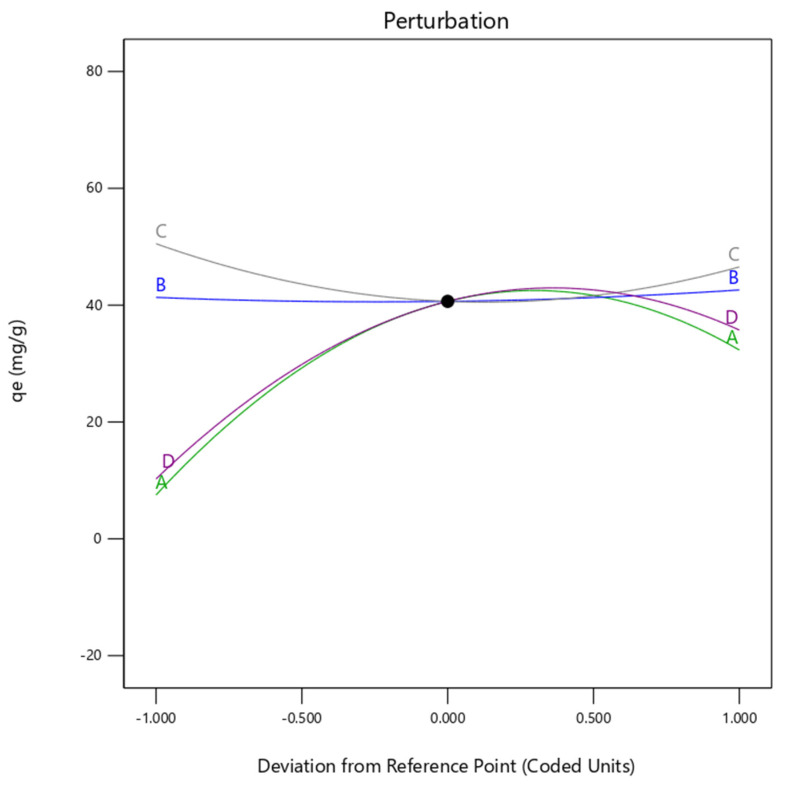
Perturbation plot of Cu(II) removal efficiency onto H^+^ Dowex-M4195 chelating resin from the selected quadratic model related to the pH, Time, Dose, Conc. and *q_e_*.

Acceptable precision was also subtracted from the measurement of the signal to noise ratio obtained from the fit statistics. The results showed that there was adequate precision of about 13.93 (>greater than 4), indicating that it can be used to navigate the design space in reasonable agreement. Therefore, the selected quadratic model (Equation (4)) can be utilized in the engineering process design to navigate the removal of divalent metals from water or wastewater treatment plants through the ion exchange method. In addition, the lack of fit F-value of about 1.76 indicates the lack of fit was not significant relative to the pure error (see Table 4). Therefore, the confidential selected quadratic model was utilized to represent the 3D dimensional plots of Cu(II) removal efficiency onto H^+^ Dowex-M4195 chelating resin related to the pH, Time, Dose, Conc. and *q_e_* as shown in Figure 13. Response *q_e_* or Cu(II) removal was computed and depended on the inputs of operating variables. The results of 3D dimension plots found that the removal capability for Cu(II) removal had an effect on all variable factors, especially pH (B) and Dose (C) as indicated in the perturbation plot (Figure 14). It can be concluded that the Cu(II) removal efficiency was affected by those variables that were related to the previous discussion above. 

### 2.10. Optimum Conditions of Cu(II) Removal at a Low pH with RSM and Model Fit

**Figure 15:**
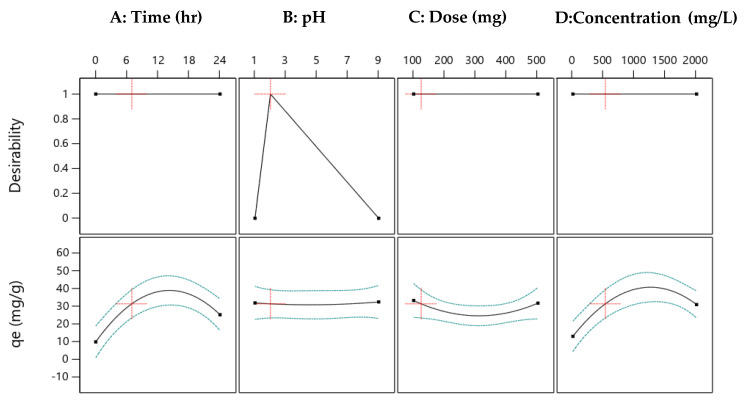
Desirability ramps of Cu(II) removal efficiency onto H^+^ Dowex-M4195 chelating resin from the selected quadratic model at a low pH.

Various water and wastewater treatments, primary testing before engineering process design and optimization using the design of experiment have been widely applied to overcome operational restrictions to achieve the highest Cu(II) removal in the treatment process. In general, a software tool (DOE) has been applied to stipulate code and actual variable factors related to observed and predicted responses of Cu(II) removal onto H^+^ Dowex-M4195 obtained in the general area of this research. The highest elimination efficiency achieved can be set as a desirable target of maximizing or minimizing, in a range or equal to the response and factor. This research focused on ion exchange at a low pH that was set as the target (pH 2) as shown in Figure 15 (B: pH). The desirabilities were designated to reflect the optimum conditions for the ion exchange method for all factors (Time, pH, Dose and Conc.) and response (*q_e_*) that were selected as 1. The optimization results found that Cu(II) removal capacity of about 31.33 mg/g was achieved, which could be obtained at a Time of 6.96 h, pH of 2, Dose of 124.13 mg and Conc. of 525.15 mg/L as illustrated in Figure 15 with the lowest pH. 

To confirm the reasonable agreements of the selected empirical quadratic model, the desirable optimum condition factor results at a low pH of 2 were substantiated or compared with the experimental results. It was found that the Cu(II) removal capacity obtained (31.65 mg/g) was similar to the selected empirical quadratic model of Equation (4) with a percent error of about 1.01, which was accepted. It can be seen that the RSM method was appropriate for the prediction Cu(II) removal capacity before the engineering process design through the ion exchange process with the selected polymeric chelating resin. 

## 3. Materials and Methods

### 3.1. Preparation of the Exchanger H^+^ Dowex-M4195 Chelating Resin 

**Figure 16:**
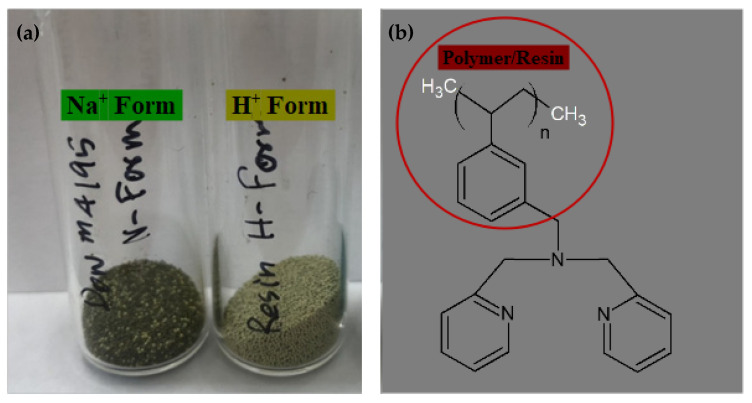
(**a**) Na^+^ form and H^+^ form Dowex-M4195 chelating resin and (**b**) the structure of polystyrene–divinylbenzene matrix, bispicolamine functional group or polymeric resin.

Raw precursor chelating resin, Dowex-M4195 (Figure 16), in this research was obtained from Dow Chemical Company, supplied by Supelco (bispicolamine functional group). Dowex-M4195 beads have a size of 3 × 10^6^ to 8 × 10^6^ angstrom and the structure is macroporous and in ionic form (Na^+^) with a 60 °C maximum temperature of 0–7 pH range. The ratio 1 (g):10 (mL) of chelating resin obtained was soaked together with 2 M of acetic acid (AR grade, Ajax Finechem, 99.99%) for over 24 h to form a hydrogen (H^+^) bond structure as illustrated in earlier research [47,48,49]. Subsequently, wet H^+^ Dowex-M4195 chelating resin form was poured several times using double distilled water to eliminate superfluous acid until the pH was about neutral and then dried in an oven at 50 °C over 12 h. The dried H^+^ form Dowex-M4195 resin was preserved and cooled down to a room temperature of around 25 °C in a glass tube for supplementary batch ion exchange investigation and optimization via response surface methodology.

### 3.2. Characterization before and after Ion Exchange for H^+^ Dowex-M4195 Chelating Resin

A surface area pore size and pore volume distribution analyzer (Bell Sorp mini, TriStar II Plus Version 3.00, Norcross, GA, USA) was used to analyze the physical properties such as the specific surface area, pore volume and pore size distribution before (H^+^ Dowex-M4195) and after (Cu(II)-loaded H^+^ Dowex-M4195 chelating resin) ion exchange for H^+^ Dowex-M4195 chelating resin. H^+^ Dowex-M4195 chelating resin before and after ion exchange was aired/degassed for over 3 h before testing the physical properties. Brunauer–Emmet–Teller (BET) and Barrett–Joyner–Halenda (BJH) approaches determined the specific surface area, total pore volume and pore size diameter. Nitrogen adsorption–desorption isotherms of H^+^ Dowex-M4195 and Cu(II)-loaded H^+^ Dowex-M4195 chelating resin were examined where the adsorption point was at 0.1–0.99 and desorption point at 0.96–0.99 within the relative pressure P/P_0_ range.

Surface morphology together with elemental analysis before and after ion exchange were examined with scanning electron microscope energy–dispersive X-ray spectroscopy (SEM-EDX) with model SEM-TM4000Plus, HITACHI. Crystal structure of the chelating resin before and after the ion exchange process was investigated using X-ray powder diffraction by measuring the intensity (counts) of radiation reflected (2 theta, coupled two theta/theta) at various angles from 5° < 2*θ* < 90° whereas registering of Cu K^α^ occurred at 1.54060. Fourier transform infrared spectroscopy (ATR-FTIR) was applied to determine changes in the structure of the surface functional groups of H^+^ Dowex-M4195 chelating resin before and after the ion exchange process at the cover wavelength range of 400–4000 cm^−1^ via a Perkin Elmer instrument analyzer with Spectrum Standard v10.4.2 software (Bangkok, Thailand).

### 3.3. RSM Relevant Statistical Analysis for Cu(II) Removal Using H^+^ Dowex-M4195 Chelating Resin Adsorbent

To find the optimal conditions for synthetic Cu(II) wastewater removal in acidic solution (pH less than 2) in a batch system via an ion exchange process, four variable factors (initial Time, pH, Dose and Concentration (Conc.) at room temperature around 25 °C) were further explored. RSM has been widely used for designing experiments to achieve the optimal response to operational factors using a design of experiment (DOE, State-Ease, Minneapolis, MI, USA, free trial) software tool. Central composite design (CCD) of the four factors was used to evaluate the advanced design using a second-degree polynomial. A total of 30 batch systems were run with 16 (2^a^ = 2^4^ = 16) factorial points, 8 (2a = 2 × 4 = 8) axial points and 6 (A_0_) central points as presented in Equation (1):

(2) $${A=2}^{a}{+2a+A}_{0}{=2}^{4}+(2\;\times \;4)+6=30,$$

where a is the number of operational factors implicated, 2^a^ indicates the number of factorial points, 2a is the number of axial points, A represents the number of batch experiments and A_0_ is the center point. After testing for 30 runs, the experimental results were determined as a second-degree polynomial regression model as exemplified in Equation (2):

(3) $${Y=\alpha }_{0}+\sum_{i=1}^{n}\alpha_{i}x_{I}+\sum_{i=1}^{n}\alpha_{\mathrm{ii}}x_{i}^{2}+\sum_{i=1}\sum_{i\neq j=1}\alpha_{\mathrm{ij}}x_{i}x_{\mathrm{ij}}+\varepsilon \;,$$

where *y* (*q_e_* mg/g) represents the response variable of observed and predicted *q_e_*, *x*_i_ and *x*_j_ are the process factors of actual or coded variables (Time, pH, Dose and Conc.) and α_0_, α_i_, α_ii_ and α_ij_ are the regression coefficients. 

Table 6 shows overall numeric factors of experimental range and levels of CCD. Each process factor was set to 5 levels: low (−alpha) and high (+alpha) levels, center point level indicated as 0 and two outer points of −1 and +1. This approach, the range and levels of CCD were duplicated for every combination of the categorical factor levels as demonstrated in Table 7.

### 3.4. Optimization via Batch Ion Exchange Studies

At research laboratory-scale, the optimization of Cu(II) was carried out using a 250 mL Erlenmeyer flask containing 100 mL synthetic Cu(II) in acidic solution. Stock solution (2000 ppm) of Cu(II) was prepared by mixing copper (II) nitrate trihydrate (Cu(NO_3_)_2_ ·3H_2_O, AR grade, Qrec, New Zealand) [24,50] in glacial acetic acid (CH_3_COOH, AR grade, Qrec, New Zealand) solution of about 0.2 M concentration [51]. The concentration of acetic acid used is that applied in biohydrometallurgy due to it being eco-friendly and inexpensive for the establishment of metallic complex solution for the ambient temperature and the pressure [52] of the surrounding air, gas or liquid in a specific location.

**Figure 17:**
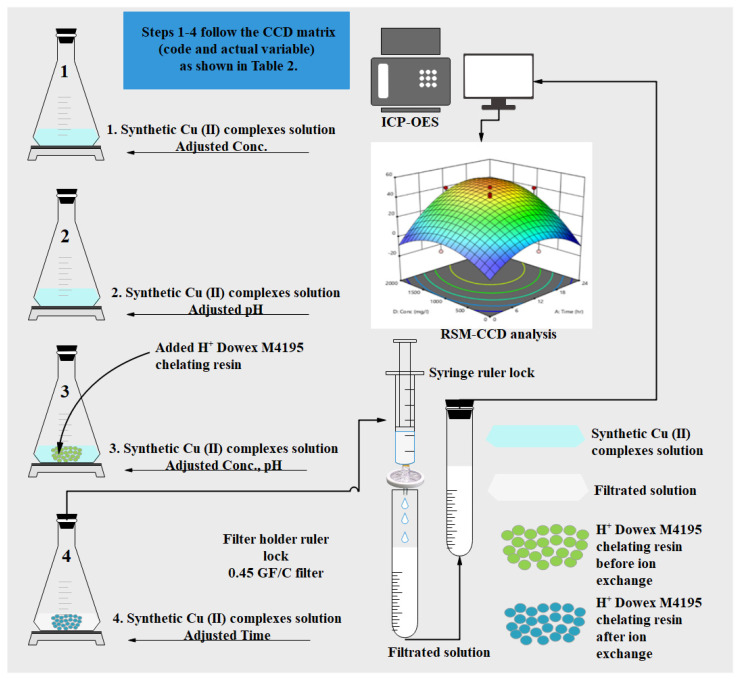
Optimization steps via batch ion exchange studies.

Batch ion exchanges were performed under the optimal conditions (pH, Concentration (Conc.), Dose and Time) to the desired level of pH (2.0 ± 0.2–9.0 ± 0.2) of each synthetic Cu(II) in acidic concentration of about 0–2000 ppm. First, 0.5 N sulfuric acid (H_2_SO_4_) and 1.0 N sodium hydroxide (NaOH) were used to adjust the pH before running the ion exchange processes. After adjusting the pH, the H^+^ Dowex-M4195 chelating resins (100–500 mg range) were added to the prepared synthetic solution at room temperature and then the response time was varied from 0–24 h. After the ion exchange processes were completed, an aliquot was withdrawn and three drops of sodium sulfite (NaSO_3_) were added to stop the reaction for each ion exchange process run, and then filtrated via a 0.45 GF/C filter using syringe filters. The filtered solution was measured using inductive coupled plasma (ICP-OES, Optima 8000, Perkin Elmer Inc., Waltham, MA, USA) at 327.393 nm wavelength for analyzing the capacity of Cu(II) adsorbed onto the H^+^ Dowex-M4195 chelating resin. The thirty-batch experiments (as shown in Table 7) and blank solution samples were run about three times to establish the trustworthiness and exactness of the experimental results, which did not exceed a maximum of about 5% for the relative standard deviations. The adsorption/ion exchange capacity of Cu(II) exchanged *q_e_* (mg/g) for each experimental run was determined with Equation (3).

(4) $$q_{e}=\frac{C_{i}-C_{e}}{m}v,$$

in which *C_i_* (mg/L) is the initial Cu(II) concentration and *C_e_* (mg/L) is the equilibrium Cu(II) adsorbed concentration, *m* (g) is the mass of the H^+^ Dowex-M4195 chelating resin, *V* (L) is the volume of metal–organic solution and *q_e_* is mg of copper (II) adsorbed per gram of chelating resin obtained. The steps of optimization via batch ion exchange experiments are shown in Figure 17.

## 4. Conclusions

The novel H^+^ Dowex-M4195 chelating resin was prepared from Na^+^ Dowex-M4195 chelating resin by using 2 M acetic acid and compared to the Cu(II)-loaded H^+^ Dowex-M4195 chelating resin. The characterization of physical and surface chemical properties before and after ion exchange was performed. The results showed that H^+^ chelating resin and Cu(II)-loaded Dowex-M4195 were mainly macroporous in nature, with a surface area of 26.5060 m^2^/g and 21.7810 m^2^/g, respectively. Additionally, H^+^ chelating resin had a high carbon content and pore volume while the functional groups showed predominantly C=O (pyridine rings) stretching vibration groups. On the other hand, Cu(II)-loaded Dowex-M4195 had a lower carbon content and pore volume than the H^+^ chelating resin and showed predominantly N=Cu, N-Cu, N-O or N=O groups, indicating Cu(II)-loaded Dowex-M4195 was effective in ion exchange between copper and resin. The physical morphology showed that before and after ion exchange, resin had a certain direction of the arrangement of tiny crystals with a low porosity, lacking a variable pore size distribution. The crystalline structure of before and after materials indicated a predominantly polymeric structure in nature. In addition, RSM and model fit for Cu(II) removal onto H^+^ Dowex-M4195 chelating resin were good, all statistical tools such as R-squared, adjusted R-squared, predicted R-squared, order polynomial, normal plot of residuals, Cook’s distance, residual vs. predicted and residual vs. run plots confirmed reasonable agreements of the selected empirical quadratic model. The optimal condition for Cu(II) removal capacity was achieved at about 31.33 mg/g, which could be obtained at a Time of 6.96 h, pH of 2, Dose of 124.13 mg and Conc. of 525.15 mg/L at the desired lowest pH. In conclusion, this study shows that modified H^+^ Dowex-M4195 chelating resin can be applied as a precursor for copper-loaded wastewater and other applications for the engineering process design of divalent metal in the electronic waste industries. 

## Figures and Tables

**Table 1:** Elemental analysis of compositions of H^+^ Dowex-M4195 and Cu(II)-loaded H^+^ Dowex-M4195 chelating resin.

Element	H^+^ Dowex-M4195 (wt %)	Cu(II)-Loaded H^+^ Dowex-M4195 (wt %)
Carbon (C)	64.50	58.51
Oxygen (O)	20.26	24.82
Nitrogen (N)	11.77	11.00
Fluorine (F)	0.38	ND
Sulfur (S)	3.01	1.01
Copper (Cu)	ND	4.66

Note: ND = Not detected.

**Table 2:** Physical properties of H^+^ Dowex-M4195 and Cu(II)-loaded H^+^ Dowex-M4195 obtained from a different method.

Method	Physical Properties	H^+^ Dowex-M4195	Cu(II)-Loaded H^+^ Dowex-M4195
BET	Specific surface area (m^2^/g)	26.5060	21.7810
	Total pore volume (cm^3^/g)	0.2892	0.2687
	Micropore volume (cm^3^/g)	LD	LD
	Mesopore volume (cm^3^/g)	LD	LD
	Macropore volume (cm^3^/g)	0.2892	0.2687
	Average pore diameter Å (angstrom)	493.6370	436.5590
BJH	Specific surface area (m^2^/g)	28.2635	24.2043
	Total pore volume (cm^3^/g)	0.2690	0.2545
	Micropore volume (cm^3^/g)	LD	LD
	Mesopore volume (cm^3^/g)	LD	LD
	Macropore volume (cm^3^/g)	0.2690	0.2545
	Average pore diameter Å (angstrom)	380.7060	420.6690

Note: LD = Less detection.

**Table 3:** Model summary statistics for Cu(II) loaded onto H^+^ Dowex-M4195 chelating resin.

Source	Sequential *p*-Value	Lack of Fit *p*-Value	Standard Derivative	R-Squared	Adjusted R-Squared	Predicted R-Squared	PRESS	
Linear	0.0055	0.0023	18.06	0.4316	0.3407	0.1624	12010.91	
2FI	0.3545	0.0023	17.66	0.5866	0.3690	−0.3035	18690.54	
Quadratic	<0.0001	0.2775	5.82	0.9645	0.9314	0.8553	2075.51	Suggested
Cubic	0.1563	0.9968	4.33	0.9921	0.9620	-	*	Aliased

Note: * cases (s) with leverage of 1.0000: PRESS statistic not defined.

**Table 4:** ANOVA results for an obtained quadratic model of Equation (4).

Source	Sum of Squares	df	Mean Square	F-Value	*p*-Value	
Model	13,830.46	14	987.89	29.13	<0.0001	significant
*x*_1_-Time	2770.33	1	2770.33	81.69	<0.0001	
*x*_2_-pH	7.24	1	7.24	0.2134	0.6508	
*x*_3_-Dose	69.06	1	69.06	2.04	0.1741	
*x*_4_-Conc.	3011.38	1	3011.38	88.79	<0.0001	
*x*_1_·*x*_2_	2.50	1	2.50	0.0736	0.7899	
*x*_1_·*x*_3_	22.47	1	22.47	0.6625	0.4284	
*x*_1_·*x*_4_	2121.52	1	2121.52	62.56	<0.0001	
*x*_2_·*x*_3_	0.0004	1	0.0004	0.0000	0.9973	
*x*_2_·*x*_4_	2.50	1	2.50	0.0736	0.7899	
*x*_3_·*x*_4_	40.30	1	40.30	1.19	0.2929	
*x*_1_^2^	1188.83	1	1188.83	35.05	<0.0001	
*x*_2_^2^	4.81	1	4.81	0.1417	0.7118	
*x*_3_^2^	120.71	1	120.71	3.56	0.0787	
*x*_4_^2^	756.00	1	756.00	22.29	0.0003	
Residual	508.72	15	33.91			
Lack of Fit	395.98	10	39.60	1.76	0.2775	not significant
Pure Error	112.73	5	22.55			
Cor Total	14,339.18	29				

**Table 5:** Code and actual variable factors related to observed and predicted responses of Cu(II) loaded onto selected H^+^ Dowex-M4195 chelating resin.

Run	Code Variable				Actual Variable					Responses *q_e_* (mg/g)		
	*x*_1_	*x*_2_	*x*_3_	*x*_4_		*x*_1_	*x*_2_	*x*_3_	*x*_4_	Observed	Predicted	Residual
										Value	Value	Value
1	−1	−1	1	1		0	1	500	2000	0.00	−2.70	2.70
2	−1	−1	1	−1		0	1	500	0	0.00	−1.25	1.25
3	0	0	−1	1		12	5	100	2000	53.20	47.18	6.02
4	0	0	0	1		12	5	300	2000	39.20	35.75	3.45
5	1	0	0	0		24	5	300	1000	39.07	32.33	6.74
6	1	1	−1	−1		24	9	100	0	0.00	1.86	−1.86
7	0	0	0	−1		12	5	300	0	0.00	10.25	−10.25
8	0	0	0	0		12	5	300	1000	40.93	40.65	0.28
9	0	0	0	0		12	5	300	1000	37.07	40.65	−3.59
10	0	0	0	0		12	5	300	1000	37.73	40.65	−2.92
11	−1	1	1	−1		0	9	500	0	0.00	−1.57	1.57
12	1	−1	−1	−1		24	1	100	0	0.00	0.58	−0.58
13	−1	0	0	0		0	5	300	1000	0.00	7.51	−7.51
14	−1	−1	−1	1		0	1	100	2000	0.00	2.02	−2.02
15	−1	1	−1	1		0	9	100	2000	0.00	3.30	−3.30
16	1	1	1	−1		24	9	500	0	0.00	−1.37	1.37
17	−1	1	−1	−1		0	9	100	0	0.00	−3.08	3.08
18	1	1	−1	1		24	9	100	2000	52.40	54.30	−1.90
19	1	−1	−1	1		24	1	100	2000	69.20	51.44	−2.24
20	−1	1	1	1		0	9	500	2000	0.00	−1.44	1.44
21	0	0	0	0		12	5	300	1000	50.13	40.65	9.48
22	1	−1	1	1		24	1	500	2000	79.76	41.98	−2.22
23	1	1	1	1		24	9	500	2000	42.88	44.82	−1.94
24	0	0	0	0		12	5	300	1000	43.07	40.65	2.41
25	0	0	0	0		12	5	300	1000	43.33	40.65	2.68
26	1	−1	1	−1		24	1	500	0	0.00	−2.63	2.63
27	0	−1	0	0		12	1	300	1000	67.04	41.34	−2.30
28	0	1	0	0		12	9	300	1000	44.13	42.61	1.53
29	−1	−1	−1	−1		0	1	100	0	0.00	−2.78	2.78
30	0	0	1	0		12	5	500	1000	39.76	46.55	−6.79

Note: *x*_1_ = Time, *x*_2_ = pH, *x*_3_ = Dose and *x*_4_ *=* Conc.

**Table 6:** Variable process factors of investigational levels of CCD.

Factor/Name	Unit	Levels (Coding Actual)		
		−1 (Low Level)	0 (Central Level)	1 (High Level)
*x*_1_, Time	h	0	12	24
*x*_2_, pH	-	1	5	9
*x*_3_, Dose	mg	100	300	500
*x*_4_, Conc.	ppm	0	1000	2000

**Table 7:** Thirty batch experiments of code and actual variable factors related to observed and predicted responses.

Run	Code Variable				Actual Variable			
	x_1_	x_2_	x_3_	x_4_	x_1_	x_2_	x_3_	x_4_
1	−1	−1	1	1	0	1	500	2000
2	−1	−1	1	−1	0	1	500	0
3	0	0	−1	1	12	5	100	2000
4	0	0	0	1	12	5	300	2000
5	1	0	0	0	24	5	300	1000
6	1	1	−1	−1	24	9	100	0
7	0	0	0	−1	12	5	300	0
8	0	0	0	0	12	5	300	1000
9	0	0	0	0	12	5	300	1000
10	0	0	0	0	12	5	300	1000
11	−1	1	1	−1	0	9	500	0
12	1	−1	−1	−1	24	1	100	0
13	−1	0	0	0	0	5	300	1000
14	−1	−1	−1	1	0	1	100	2000
15	−1	1	−1	1	0	9	100	2000
16	1	1	1	−1	24	9	500	0
17	−1	1	−1	−1	0	9	100	0
18	1	1	−1	1	24	9	100	2000
19	1	−1	−1	1	24	1	100	2000
20	−1	1	1	1	0	9	500	2000
21	0	0	0	0	12	5	300	1000
22	1	−1	1	1	24	1	500	2000
23	1	1	1	1	24	9	500	2000
24	0	0	0	0	12	5	300	1000
25	0	0	0	0	12	5	300	1000
26	1	−1	1	−1	24	1	500	0
27	0	−1	0	0	12	1	300	1000
28	0	1	0	0	12	9	300	1000
29	−1	−1	−1	−1	0	1	100	0
30	0	0	1	0	12	5	500	1000

## Data Availability

Not applicable.

## Sample Availability

Samples of the compounds are not available from the authors.
